# Low T3 State Is Correlated with Cardiac Mitochondrial Impairments after Ischemia Reperfusion Injury: Evidence from a Proteomic Approach

**DOI:** 10.3390/ijms161125973

**Published:** 2015-11-06

**Authors:** Francesca Forini, Nadia Ucciferri, Claudia Kusmic, Giuseppina Nicolini, Antonella Cecchettini, Silvia Rocchiccioli, Lorenzo Citti, Giorgio Iervasi

**Affiliations:** 1 Consiglio Nazionale delle Ricerche, Institute of Clinical Physiology, Via Giuseppe Moruzzi 1, Pisa 56124, Italy; nucciferri@ifc.cnr.it (N.U.); kusmic@ifc.cnr.it (C.K.); nicolini@ifc.cnr.it (G.N.); silvia.rocchiccioli@ifc.cnr.it (S.R.); lorenzo.citti@ifc.cnr.it (L.C.); iervasi@ifc.cnr.it (G.I.); 2 Fondazione Toscana Gabriele Monasterio, Via Giuseppe Moruzzi 1, Pisa 56124, Italy; 3 Department of Clinical and Experimental Medicine, University of Pisa, Via Volta Pisa 56124, Italy; acecch@biomed.unipi.it

**Keywords:** low T3 state, ischemia/reperfusion, mitochondrial function, mitochondria proteomic profiling

## Abstract

Mitochondria are major determinants of cell fate in ischemia/reperfusion injury (IR) and common effectors of cardio-protective strategies in cardiac ischemic disease. Thyroid hormone homeostasis critically affects mitochondrial function and energy production. Since a low T3 state (LT3S) is frequently observed in the post infarction setting, the study was aimed to investigate the relationship between 72 h post IR T3 levels and both the cardiac function and the mitochondrial proteome in a rat model of IR. The low T3 group exhibits the most compromised cardiac performance along with the worst mitochondrial activity. Accordingly, our results show a different remodeling of the mitochondrial proteome in the presence or absence of a LT3S, with alterations in groups of proteins that play a key role in energy metabolism, quality control and regulation of cell death pathways. Overall, our findings highlight a relationship between LT3S in the early post IR and poor cardiac and mitochondrial outcomes, and suggest a potential implication of thyroid hormone in the cardio-protection and tissue remodeling in ischemic disease.

## 1. Introduction

Acute myocardial infarction (AMI) leading to ischemic heart disease is a major cause of death worldwide. Although timely reperfusion effectively reduces short-term mortality, restoration of blood flow also leads to ischemia/reperfusion (IR) injury which in the long run prompts adverse cardiac remodeling. Thus, limiting cardiac damage in the early stages of the wound healing process is a critical step to improve patient prognosis.

Mitochondrial dysfunction is a key pathogenic event in cardiac IR and disease progression [1,2,3]. As a consequence, preserving mitochondrial function is essential to limit myocardial damage in ischemic heart disease. The main mechanisms for post IR mitochondrial dysfunction include impairment in electron transport chain (ETC) complex activities [4,5], defects in supermolecular assembly of ETC complexes [6,7,8], impaired ionic homeostasis and formation of reactive oxygen species (ROS) [9], and impaired tricarboxylic acid (TCA) cycle anaplerosis, [10,11]. In light of this complex scenario, protein profiling has emerged as a powerful tool allowing for simultaneous measurement of the levels of many mitochondrial proteins in a single analysis [12,13,14]. Moreover, analyses of the mitochondrial proteome prove useful not only to expand our knowledge of mitochondrial function in physio/pathological states, but also to unveil potential strategies for therapeutic intervention [15].

Triiodothyronine (T3), the biologically active thyroid hormone (TH), is considered a major regulator of mitochondrial activity [16,17,18]. The breakdown of thyroid system homeostasis is associated with bioenergetic remodelling of cardiac mitochondria which leads to severe alterations in the biochemistry, structure and contractility of cardiac muscle [17]. Clinical evidence shows post IR declines of T3, known as low T3 state (LT3S) [19,20,21] that represent a strong independent prognostic predictor of death and major adverse cardiac events [22]. Accordingly LT3S correction exerts cardioprotective actions both in the clinical arena and in animal models of post ischemic cardiac diseases [23,24,25]. Since mitochondria are critical effectors of the T3 cardioprotective signaling [26,27], characterization of the mitochondrial proteome remodeling in the post IR-LT3S model may reveal candidate proteins and pathways related to this signaling.

The main objective of the present study is to compare the post IR rat mitochondrial proteome in the early phase of wound healing between normal and low T3 states. Differentially expressed proteins were assessed in comparison to sham operated animals. Our data indicate that mitochondrial proteomic alteration and dysfunction are mainly associated with a post IR decrease of T3 levels.

## 2. Results

### 2.1. Validation of the Post IR Low-T3 State (LT3S) Model

We have previously observed that in our AMI model, cardiac TH concentrations closely reflect those of the circulating free TH [27,28]. Therefore, in the present study the circulating levels of free T3 (FT3) and free T4 (FT4) were assessed at baseline and 3 days (3d) post-surgery to track the myocardial TH status. As summarized in Table 1, sham operation did not significantly change serum TH concentrations. No significant alterations of 3d FT3 or FT4 were measured even in IR-NT3 group, while a significant reduction of FT3 concentration was observed three days post-surgery in IR-LT3S rats. This difference should not be attributed to different degree of LV damage since all ischemia-injured rats exhibited the same damage score as well as comparable percentage of area at risk (AAR) (Table 1). These findings confirmed the previous observation about the occurrence of a L-T3S in a subset of animals in the early post-ischemic setting [27]. In accordance with clinical data, the L-T3S condition was observed in about 30% of ischemia-reperfused rats with matching degrees of damaged myocardium, thus suggesting that the Low-T3S rat may represent a useful preclinical model.

### 2.2. Post IR Myocardial Functional Parameters and Mitochondrial Activity

**Figure 1:**
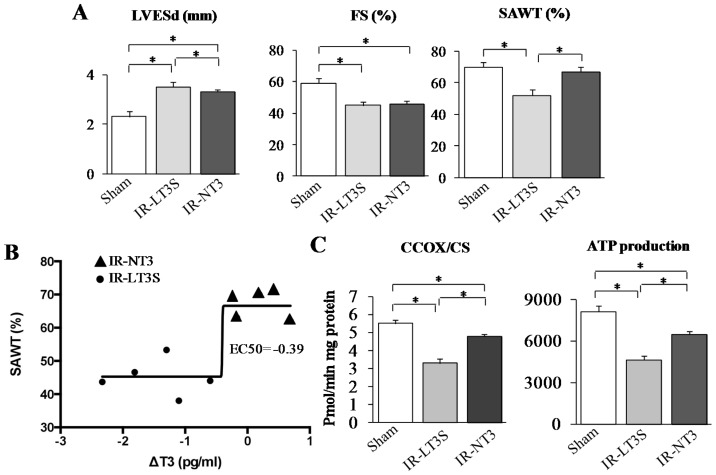
Effect of L-T3S on cardiac functional recovery and mitochondrial activity 72 h post IR. (**A**) LV end systolic diameter (LVESd) (**left** panel), fractional shortening (FS) (**medium** panel) and systolic anterior wall thickening (SAWT) (**right** panel); (**B**) Non linear fitting (sigmoid) relating the ∆FT3 values (difference between 3 days post IR and basal) to systolic anterior wall thickening (SAWT) in IR-LT3 and IR-NT3; and (**C**) mitochondrial function assessed in the LV area at risk (AAR): citrate synthase (CS) normalized cytochrome *c* oxidase (CcOx) activity (**left** panel) and ATP production (**right** panel). Data are expressed as mean ± SE; *n* = 5 in each group; * *p* ≤ 0.005.

As a second step, we asked if the observed FT3 decrease in the L-T3S group could be associated with an impaired recovery of post-ischemic cardiac function and chamber geometry. Although both groups of infarcted rats showed similar alterations of the LV fractional shortening (FS), and the end systolic LV diameter (Figure 1A), only the IR-LT3 group exhibited a significant reduction of the systolic anterior wall thickening (SAWT) with respect to both sham and IR-NT3 group (Figure 1A) suggesting that a post IR L-T3S is associated with a significant reduction in regional contractility of LV within the AAR. To strengthen the hypothesis of a relationship between the variation of T3 levels (three days after IR with respect to the basal level) and the cardiac functional parameter SAWT, a non linear regression (sigmoid model) was applied to derive the EC_50_, *i.e.*, the delta T3 concentration that provokes a response half way between the minimal response (bottom point) and the maximal response (top point) (Figure 1B). The derived EC_50_ was equal to −0.39, a value that falls between LT3S and NT3 groups, and may be considered a clear cutoff of the delta T3 level.

**Table 1:** Serum free thyroid hormone and LV damage index.

TH Level (pg/mL)	Sham		IR-NT3		IR-LT3S	
	Mean ± SEM	Median (IQR)	Mean ± SEM	Median (IQR)	Mean ± SEM	Median (IQR)
FT3 basal	3.2 ± 0.3	3.6 (2.7–3.8)	3.2 ± 0.3	3.2 (2.6–3.9)	3.5 ± 0.3	3.4 (3.3–3.6)
FT3 final	3.5 ± 0.2	3.8 (3.1–3.8)	3.3 ± 0.3	3.6 (2.6–3.6)	2.2 ± 0.3 *^,#,†^	2.3 (1.9–2.4) ^§,&,+^
FT4 basal	12.4 ± 0.6	13.4 (11.6–13.7)	13.0 ± 0.7	13.3 (12.9–13.7)	12.1 ± 1.0	13.7 (13.5–14.3)
FT4 final	12.1 ± 1.5	12.6 (10.9–13.8)	14.3 ± 1.8	14.3 (12.7–16.3)	13.3 ± 1.6	10.4 (10.1–10.8)
**Damage Index**	**Sham**		**IR-NT3**		**IR-LT3S**	
	**–**		**Mean ± SEM**		**Mean ± SEM**	
Arrhythmic severity score	NA		3.3 ± 0.4		3.4 ± 0.3	
Area at risk (% of LV)	NA		48 ± 3		47 ± 5	

Free T3 (FT3) and free T4 (FT4) were measured before and 72 h post-IR; *n* = 5. Left columns report mean values ± SEM: * *p* = 0.006 *vs.* respective basal levels; ^#^ *p* = 0.004 *vs*. sham-operated; ^†^ *p* = 0.009 *vs.* IR-NT3. Right columns report median values and interquartile range (IQR), along with the corresponding non parametric analysis: ^§^ *p* = 0.04 *vs.* respective basal levels; ^&^ *p* = 0.009 *vs.* sham-operated; ^+^ *p* = 0.014 *vs.* IR-NT3. Arrhythmic score was calculated based on ECG data obtained from ischemia to 30 min of reperfusion. Area at risk measured at 72 h post IR is expressed as % of LV. NA = Not Applicable.

To evaluate if this alteration can be associated with a greater degree of mitochondrial impairment in the AAR of L-T3S rats, we next determined cytochrome *c* oxidase activity and ATP production. As shown in Figure 1C, both ischemia injured groups showed reduced citrate synthase-normalized cytochrome c activity, as well as reduced rate of ATP production, but the lowest level were in any case assessed in the L-T3S rats. These findings indicate that a decreased post IR T3 level is associated with poorer mitochondrial activity and energy production.

### 2.3. Mitochondrial Proteome

**Figure 2:**
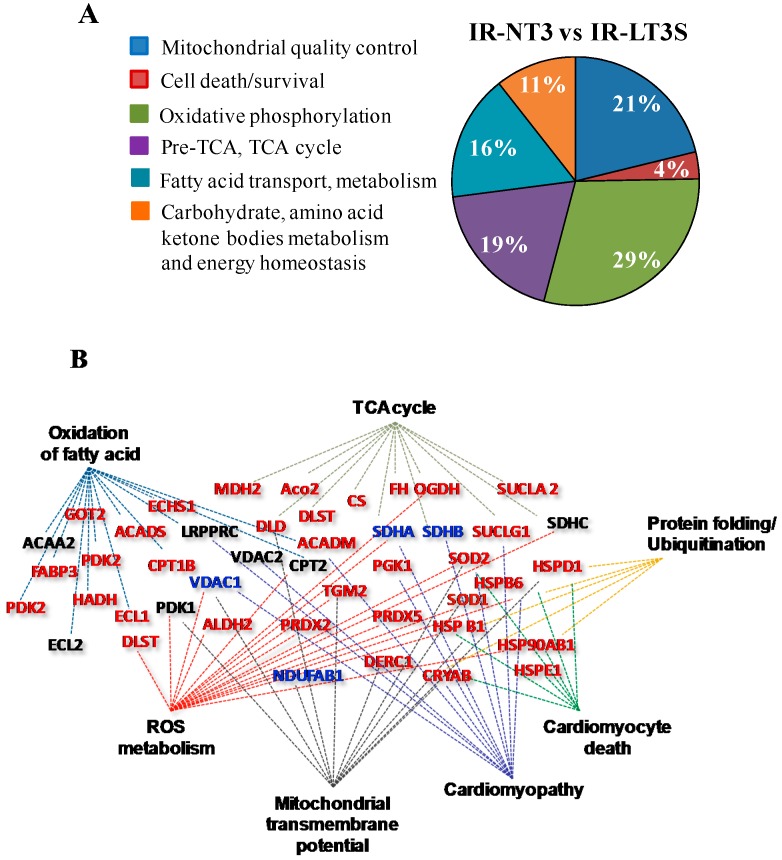
Mitochondrial proteomic analysis obtained at 72 h post IR. (**A**) Pie chart showing percentage of differentially expressed proteins grouped according to their function in IR-NT3 *vs.* IR-LT3; and (**B**) clustering of differentially expressed proteins in IR-LT3S *vs.* IR-NT3 generated by IPA software. Networks related to diseases, functions and canonical pathways were generated based on the information stored in IPA Knowledge base. Network nodes are named by correspondent Gene Codes. The color assigned to node name indicate the level of proteins expression: red for up-regulated, blue for down-regulated and black for no change in IR-NT3 *vs.* IR-LT3S respectively. Gene acronyms are listed in the abbreviation list.

A proteomic study was then performed to assess if the physiological and biochemical differences observed between IR-LT3S and IR-NT3 rats might be related to quantitative changes in the cardiac mitochondrial proteome. To this end, mitochondrial protein profiling from sham, IR-NT3 and IR-LT3S rats were obtained. The principal mitochondrial proteins were identified, as shown in the Supplementary Material (Figure S1). Multiple comparisons were performed to identify differentially expressed proteins. Of the total 546 identified proteins, 138 mitochondrial proteins exhibited significant changes and were grouped according to their function using the published literature and Uniprot database (Nucleic Acids Res. 43:D204-D212, 2015). Figure 2A shows the percentage representation of different protein groups/functions (clusters) significantly changed between IR-LT3S and IR-NT3. Twenty-five percent of altered proteins are implicated either in mitochondrial quality control (21%) or in cell death (4%). It is particularly notable that the remaining 75% belongs to functional groups that are involved in ATP synthesis.

**Figure 3:**
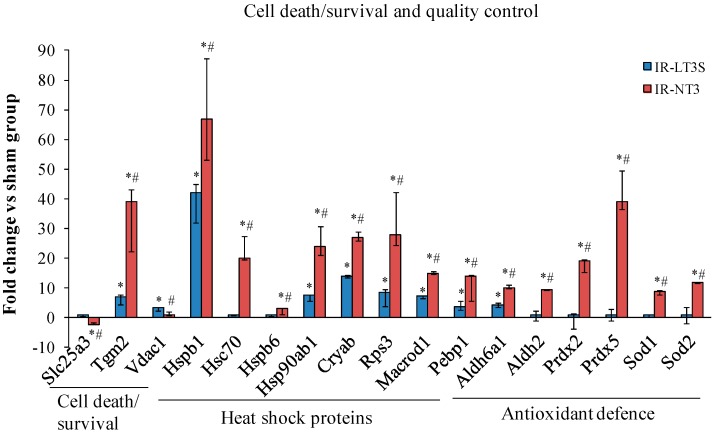
Differentially expressed proteins involved in cell death and mitochondrial quality control in response to stress. Data are expressed as median and interquartile range. * *p* *vs.* sham < 0.017; # *p* *vs.* IR-LT3S < 0.017. Protein acronyms are listed in the abbreviation list.

**Figure 4:**
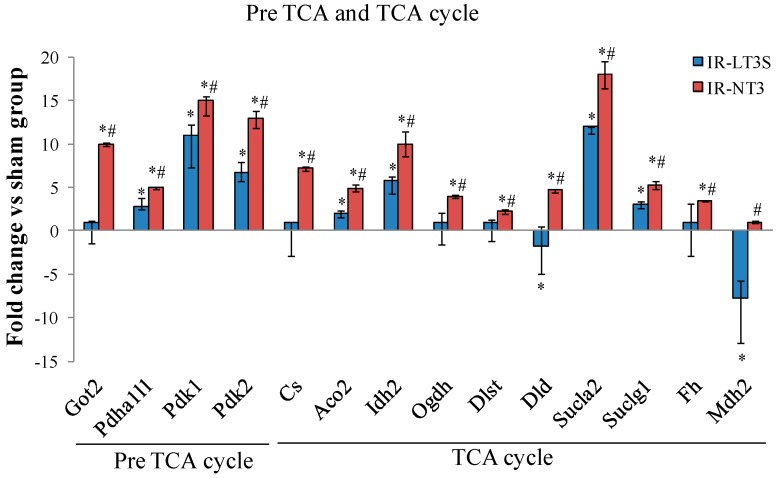
Differentially expressed proteins involved in TCA cycle and pre TCA cycle. Data are expressed as median and interquartile range. * *p* *vs.* sham < 0.017; # *p* *vs.* IR-LT3S < 0.017. Protein acronyms are listed in the abbreviation list.

**Figure 5:**
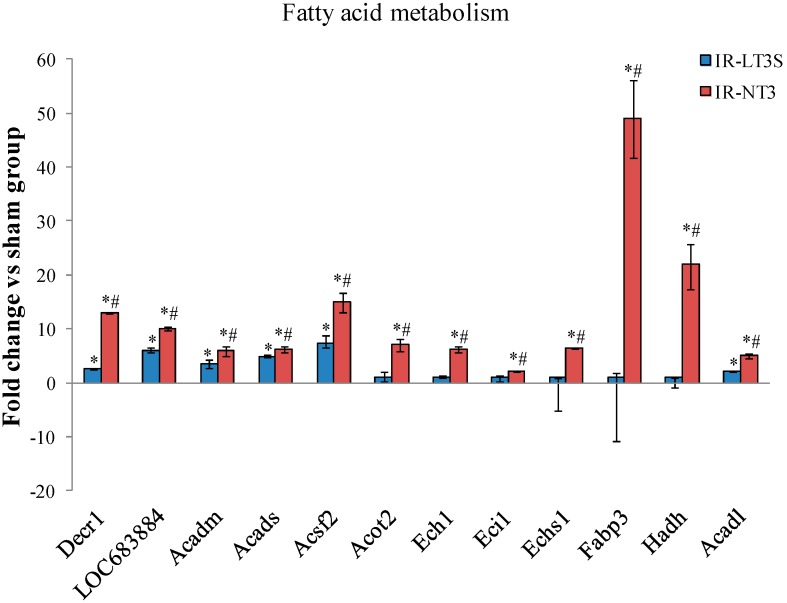
Differentially expressed proteins involved fatty acid metabolism. Data are expressed as median and interquartile range. * *p* *vs.* sham < 0.017; # *p* *vs.* IR-LT3S < 0.017. Protein acronyms are listed in the abbreviation list.

**Figure 6:**
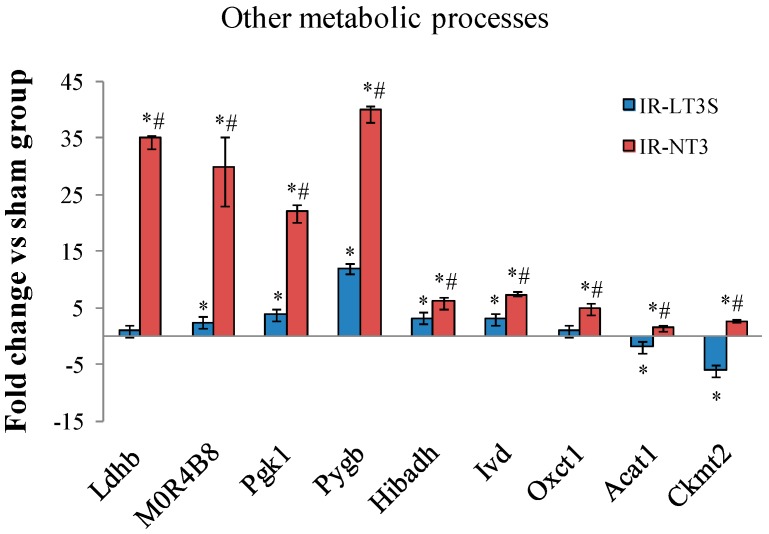
Differentially expressed proteins involved in other cellular energy metabolic processes. * *p* *vs.* sham < 0.017; # *p* *vs.* IR-LT3S < 0.017. Protein acronyms are listed in the abbreviation list.

Ingenuity Pathway Analysis (IPA, http://www.ingenuity.com/products/pathways_analysis.html, Qiagen, Venlo, Holland) was used to confirm the functional protein grouping of differentially expressed proteins in IR-NT3 *vs.* IR-LT3S and to relate them to disease. As shown in Figure 2B, the protein clusters play critical roles in mitochondrial activity and dysfunction, and in disease etiopathology (cardiomyopathy). Selected proteins from each functional group are reported in Figure 3, Figure 4, Figure 5 and Figure 6 and described below (Tables S1–S5 for the complete list).

### 2.4. Mitochondrial Quality Control and Cell Death

IR induced a significant upregulation of stress-responsive proteins (Figure 3). Notably, IR-NT3 rats exhibited the highest level of heat shock proteins (HSP), including HSP27, HSP71, HSP90 and α-crystallin (Cryab) along with a greater increase of DNA-repair-associated proteins (40s ribosomal protein S3, Rps3; and *O*-acetyl-ADP-ribose deacetylase, Macrod1) (Figure 3). By considering ROS scavenging system, six antioxidant enzymes were found to be upregulated in IR-NT3 group *vs.* both sham and IR-LT3S groups, including isoforms of aldehyde deidrogenase (Aldh6a and Aldh2), peroxiredoxines (Prdx2 and Prdx5) and superoxide dismutases (Sod1 and Sod2) (Figure 3). On the contrary, in the IR-LT3S group the level of the antioxidant enzymes was comparable to the sham group (Figure 3).

IR also differently affected proteins involved in mitochondrial-mediated cell death/survival processes. In particular, in the IR-NT3 group the phosphate carrier (Slc25a3) was downregulated *versus* both the sham and the IR-LT3S. This protein remained unchanged in the IR-LT3S rats with respect to the sham group. The increase of voltage dependent anion selective channel protein 1 (VDAC1) observed in IR-LT3S was prevented in IR-NT3 group. The protective transglutaminase 2 (TGM2) was upregulated in both IR groups, with the highest values shown by IR-NT3 rats (Figure 3).

Overall these data suggest that mitochondria of IR-NT3 rats possess higher protein quality control and greater defensive capacity to face IR injury.

### 2.5. Cellular Energy Metabolism

In accordance with well known post IR cardiac metabolic impairment, protein expression in IR rats was altered at crucial points in cellular energy metabolism.

Pre-TCA and TCA cycle enzyme isoforms were upregulated in response to IR, but the highest levels were measured in the IR-NT3 rats (Figure 4). The main modulated proteins are involved in: (1) anaplerotic reaction and malate/aspartate shuttle (aspartate aminotransferase, Got2); (2) regulation of pyruvate entry through TCA (pyruvate dehydrogenase alpha 1/1, Pdha1/1; and pyruvate dehydrogenase kinase 1 and 2, Pdk1 and Pdk2); (3) TCA cycle (citrate synthase, CS; aconitate hydratase Aco2; isocitrate dehydrogenase (NADP) Idh2; and fumarate hydratase, Fh); and (4) substrate level phosphorylation (E2 and E3 component of the 2-oxoglutarate dehydrogenase Dlst and Dld; and succinyl-CoA synthetase, Sucla 2 and Suclg1) (Figure 4).

Similarly to pre-TCA and TCA, IR induced an increase of protein subunits involved in fatty acid metabolism (Figure 5). The highest levels were found in IR-NT3 rats and the most highly expressed isoforms were involved in: (1) β oxidation and lipid biosynthesis (2,4-dienoyl CoA reductase 1, Decr1; acetyl-coenzyme A dehydrogenase medium and short chain, Acadm and Acads; δ(3,5)-δ(2,4)-dienoyl-CoA isomerase, Ech1; enoyl-CoA hydratase, Echs1; hydroxyacyl-coenzyme A dehydrogenase, Hadh; and acyl-CoA synthetase family member 2, Acsf2); (2) regulation of intracellular levels of acyl-CoAs free fatty acids and CoASH (Protein Acot 13, LOC683884); and (3) regulation of fatty acid transport and fatty acid substrate utilization (fatty acid-binding protein, Fabp3; and acyl-CoA thioesterase 2, Acot2) (Figure 5).

IR influenced also the content of several enzymes regulating other metabolic reactions (Figure 6). As for TCA and fatty acid oxidation, the highest levels were measured in IR-NT3 group. Among them, of particular interest for the post IR functional recovery are those involved in lactate metabolism and clearance, glycolysis and glycogenolysis (l-lactate dehydrogenase B chain, Ldhb; pyruvate kinase, MOR4B8; phosphoglycerate kinase 1, Pgk1; and glycogen phosphorylase, Pigb) (Figure 6). Finally, IR-NT3 rats exhibited increased levels of the mitochondrial creatine kinase isoform (CKmt2), a protein that was more severely decreased in IR-LT3S group and that plays a critical role in energy homeostasis. (Figure 6).

In line with ATP production data, these findings suggest that, with respect to IR-LT3S rats, IR-NT3 rats present a greater ability to provide metabolic intermediates for TCA and to oxidize FFA, glucose and glycogen in order to sustain greater ATP production.

## 3. Discussion

The main results of the present study is that the recovery of the pre-ischemic FT3 levels in the early period of the post IR wound healing process, is associated with better cardiac functional recovery and lower mitochondrial activity impairments in the injured LV myocardium. Moreover, proteomic profiling in IR-NT3 and IR-LT3S rats revealed a different modulation of mitochondrial proteins critically involved in the regulation of mitochondrial activity and cardiomyocyte survival, as suggested by IPA (see Figure 2B). The main proteomic results are discussed below according to the functional protein grouping.

### 3.1. Mitochondrial Quality Control and Mitochondrial-Mediated Cell Death

#### 3.1.1. Mitochondrial Quality Control

Mitochondrial reactive oxygen species (ROS) production following IR leads to extensive damage to different types of mitochondrial molecules resulting in mitochondrial dysfunction [29]. Different strategies of mitochondrial quality control have evolved to counteract the adverse effects resulting from oxidative stress [30]. Among these processes, repair of damaged molecules, refolding of misfolded proteins, ROS scavenging and removal of excessively damaged mitochondria play a key role.

Protein folding and repair are regulated by specialized proteins, termed chaperones, which include heat-shock proteins (HSPs) [31]. The protective functions of HSP70, HSP90, HSP 27 and α-crystallin B against IR injury was extensively investigated in previous studies using transgenic animals and isolated cardiac myocyte-derived cells [32,33,34]. Similarly, overexpression of the small HSPs α-crystallin B and HSP27 diminished the reversible damage after simulated or myocardial ischemia [35,36,37].

Accordingly, in our study, the post IR retention of physiological T3 levels was associated with a higher content of HSP71, HSP90, HSP27 and α-crystallin B, which was paralleled by preserved mitochondrial function.

Mitochondrial DNA is another target of IR. Mitochondrial DNA damage plays a key role in post IR disease progression, which highlights the importance of efficient repair machinery [38]. In our model of IR, two proteins implicated in DNA damage repair namely 40s ribosomal protein S3 (rpS3) and *O*-acetyl-ADP-ribose deacetylase (MACROD1) were found to be up-regulated in IR-NT3 group with respect to IR-LT3S. These proteins act synergistically through different modes of action to afford cardioprotection [39,40,41].

Antioxidant defenses are another important class of mitochondrial quality control molecules that were differentially modulated by post IR FT3 levels. Among them, Aldh2 protects the heart against ischemic injury through detoxification of toxic aldehyde and a differential regulation of autophagy [42,43], while peroxiredoxins and superoxide dismutases play a key cardioprotective role through oxide detoxification [44,45,46,47].

We propose that the higher level of quality control proteins observed in the IR-NT3 group promotes better repair of post IR mitochondrial damage, which is essential for preserved mitochondrial activity.

#### 3.1.2. Cell Fate

IR-induced mitochondrial impairments favor the formation of mitochondrial pores that open in a process known as mitochondrial permeability transition leading to apoptosis and necrosis. Inhibition of this process is cardioprotective in both patients and animal models [48,49]. In our study, the two mitochondrial permeability transition activators (namely, the phosphate carrier and Vdac1) were less expressed in the IR-NT3 group than in IR-LT3S. These data are in line with our previous observations [27] that LT3S correction by T3 replacement in the post IR setting limits mitochondrial membrane depolarization and cell death and reinforces the hypothesis of a key role of post-IR T3 levels in cardiac recovery.

When mitochondrial injuries overwhelm molecular repair capacity and antioxidant defenses, removal of damaged mitochondria through mitophagy is a protective strategy to avoid cell death. Our proteomic profiling showed in the IR-LT3 group the highest level of tissue tranglutaminase 2 (Tgm2), a cardioprotective effector that participates in the maintenance of the intact mitochondrial respiratory function and in the clearance of damaged mitochondria [50,51].

### 3.2. Oxidative Phosphorylation

The oxidative phosphorylation system (OXPHOS) in the mitochondrial inner membrane carries out the central biological process of cardiac energy metabolism. Thus, the alteration of major OXPHOS proteins is responsible for modifying all the cardiac energy metabolism and performance. As expected, we found in both injured groups a significant dysregulation of all ETC and ATP synthesis complexes, the lowest levels being measured in IR-NT3 group. The apparently contradictory result of lower OXPHOS protein levels in the presence of better preserved functional recovery and mitochondrial activity observed in the IR-NT3 group, may have several explanations. First, with the exception of complex II, all ETC complexes can associate themselves in supercomplexes, known as respirasomes, that organize electron flux to optimize the use of available substrates [52]. Mitochondrial defects can arise from supramolecular assembly rather than from the individual components of the ETC [6]. We speculate that the higher level of quality control proteins in IR-NT3 rats guarantees the assembly of intact, correctly-folded mitochondrial components in more functional macromolecular complexes, which may explain higher ATP production in the presence of lower ETC protein content. Second, post-translational modifications (PTMs) have emerged as powerful regulators of mitochondrial function and in particular of the mitochondria-encoded subunit 1 of the complex IV [53,54,55]. We might speculate that different post IR T3 levels may have induced different post-translational modifications (PTMs) in ETC complexes. Our proteomic approach was not intended to analyze PTMs, further dedicated studies are needed to explore this critical issue.

### 3.3. Pre TCA, TCA Cycle

The tricarboxylic acid cycle (TCA) forms a major metabolic hub and as such it is involved in many disease states involving energetic imbalance. In hypoxic conditions such as in the post IR setting, when OXPHOS is impaired, the TCA supplies high-energy phosphates through matrix substrate-level phosphorylation catalyzed by the succinyl coenzyme A synthetase [56,57].

It has been demonstrated that reduction of glycogen turnover and depletion of TCA substrates contributes to impaired contractile function of ischemia/reperfused myocardium, and that TCA intermediates, along with essential substrates, such as glucose, lactate, and pyruvate, are necessary to ensure functional recovery with reperfusion. [58,59]. A central role in substrate level phosphorylation is played by α-ketoglutarate dehydrogenase, that supply succinyl-CoA to succinyl coenzyme A synthetase, and by malate-aspartate shuttle, that is involved in anaplerotic supply of substrate to TCA [56].

In our study the decreased OXPHOS protein content following the ischemic injury was counterbalanced by an increase in pre-TCA, and TCA cycle enzymes, as well as in glycolytic enzymes. This tendency was more evident in the presence of preserved post IR FT3 and in particular regarded the enzymes involved in anaplerotic reaction and in the substrate level phosphorylation. These data suggest that the preserved TH level may play a key role in favoring mitochondrial anaerobic production of ATP. Our data are in agreement with previous findings in multiple animal models showing that T3 supplementation modulates pyruvate entry into the TCA, thereby providing the energy support for improved cardiac function after reperfusion [60,61]. We speculate that the effects of preserved physiological levels of T3 on α-ketoglutarate dehydrogenase might improve post IR cardiac efficiency. Indeed, supply of α-ketoglutarate during blood cardioplegia attenuated ischemic injury in patients undergoing coronary operations [62,63]. Similarly, T3 administration in patients with L-T3S induced by cardiopulmonary bypass improved postoperative ventricular function, reduced the need for treatment with inotropic agents and mechanical devices, and decreased the incidence of myocardial ischemia [64].

### 3.4. Fatty Acids Metabolism

In healthy hearts, >70% of the cardiac energy is accounted for by oxidation of fatty acids (FAs) and the remainder by glucose oxidation. However, the heart changes its substrate preference from FAs towards glucose as remodeling develops in response to diverse stresses including IR [65]. This metabolic shift may be an adaptive mechanism under acute stress condition such as IR, because it lowers oxygen consumption. On the other hand, glucose oxidation yields far less ATP than FAs. Insufficient ATP production is likely to increase the susceptibility of post-infarct hearts to cardiomyocyte death and contractile dysfunction [66]. Accordingly, reversal of metabolic shift in post IR remodeling markedly improved contractile function [67].

Here we report that retention of physiological post IR T3 levels in the early post IR phase is associated with the upregulation of several proteins involved in FA oxidation.

If confirmed through mechanistic inferences, these data might support and extend to cardiovascular pathologies, such as IR, the notion that changes of TH levels affect the myocardial mitochondrial bioenergetic capacity [68].

### 3.5. Study Limitations and Concluding Remarks

The results, obtained in a small population of animals, are associative and, in the absence of an intervention group (for example ischemia/reperfusion animals treated with T3), at present we are unable to infer any cause and effect relationship neither between post IR T3 level and cardiac/mitochondrial function, nor between proteomic remodeling and cardiac impairment. Moreover, the assumption that circulating T3 levels are a substitute of myocardial T3 content derives from data of previous studies [27,28] rather than a direct measurement in the present study.

Nevertheless, our data clearly indicate specific changes in mitochondrial protein expression in relation to different post IR circulating T3 level. Retention of physiological T3 concentration is associated with the upregulation of proteins with functional relevance in rescue of mitochondrial integrity and in optimization of substrate utilization. These differences along with the better recovery of post IR cardiac function and mitochondrial activity in the NT3 rats prompt us to speculate that a condition of L-T3S in the early setting of the post IR wound healing might affect mitochondrial function and contribute to adverse remodeling.

## 4. Material and Methods

### 4.1. Animal Procedure

**Figure 7:**
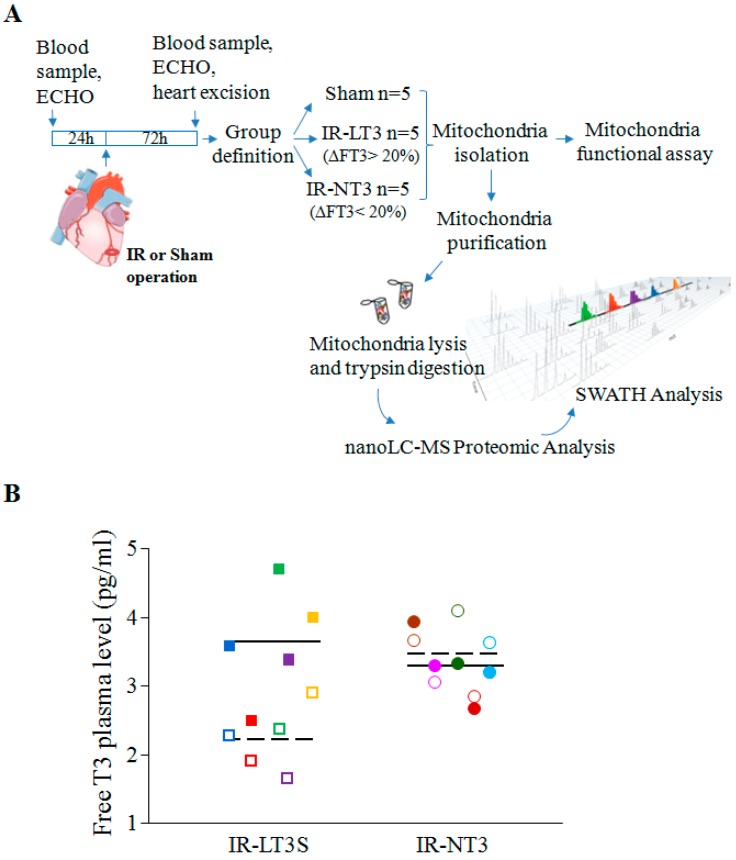
(**A**) Flow chart showing the study design. Seventy-two hours post IR, five rats developed a low T3 state (IR-LT3S) while 12 maintained normal FT3 plasma levels (IR-NT3 group). All five rats of the IR-LT3S group were used, and for a balanced statistical analysis between groups of equal numbers, five were randomly enrolled out of the 12 IR-NT3 rats; (**B**) Scatter-plot showing FT3 plasma levels in the IR-LT3S group (squares) and NT-3 group (circles) at baseline (filled symbols) and three days post IR (empty symbols). Different colors represent different rats. Continuous and dashed lines indicate the mean values before and after IR respectively.

The study was performed in accordance with the European Directive (2010/63/UE) and the Italian law (D.L 26/2014), and the protocol was approved by the Animal Care Committee of the Italian Ministry of Health (Endorsement n.240/2011-B, 9 November 2011). All surgery was performed under anaesthesia, and all efforts were made to minimize suffering. The study design is depicted in the flow chart reported in Figure 7. A total of 24 male Wistar rats were used in the study. Five rats were assigned in the sham group and 19 underwent IR. Out of 19 animals subjected to LAD occlusion, two died during surgery due to irreversible ventricular fibrillation following the IR protocol.

Myocardial infarction and reperfusion was produced by 30 min ligation of the left descending coronary artery (LAD) followed by reperfusion of adult male Wistar rats 12–15 weeks old and weighing 310 ± 3 g using a technique described in detail elsewhere [27]. A standard limb D1–D3 electrocardiogram (ECG) was continuously monitored during surgery up to 60 min.

In all cases ischemia was confirmed by ST segment elevation in the ECG and visually assessed regional cardiac cyanosis. In addition, occurrence of arrhythmias both during ischemia and at reperfusion was also recorded: arrhythmias were classified according to the Lambeth Conventions. A scoring system was used to classify their severity as previously described [69].

The severity score of ventricular arrhythmias was used as index of ischemia damage and a score level equal or greater than three was adopted as inclusion criteria in the study. A group of sham-operated rats was used as control. Three days after surgery hearts were arrested in diastole by a lethal KCl injection. Cardiac tissue samples were obtained from the core of the ischemic reperfused region (area at risk, AAR) of LV as describe elsewhere [27]. In sham-operated animals tissues were harvested from corresponding regions. Samples from each area were immediately processed for mitochondria isolation. Rats undergone I/R were allocated to two groups according to the percent decrease of T3 circulating levels measured at 72 h after surgery against the basal value. In particular, a reduction ≥20% of the basal level of T3 measured 3 days after surgery was used as cut-off value to enroll animals in the low T3-state group (IR-LT3S). All the other rats were included in the normal T3 group (IR-NT3).

### 4.2. Echocardiography Study

Echocardiographic studies were performed three days after infarction with a portable ultrasound system (MyLab 25, Esaote SpA, Genova, Italy) equipped with a high frequency linear transducer (LA523, 12.5 MHz). Images were obtained from the sedated animal, from the left parasternal view. Short-axis two-dimensional view of the left ventricle (LV) was taken at the level of papillary muscles to obtain M-mode recording. Anterior (ischemia-reperfused) and posterior (viable) end-diastolic and end-systolic wall thicknesses, systolic wall thickening, and LV internal dimensions were measured following the American Society of Echocardiography guidelines. Parameters were calculated as mean of the measures obtained in three consecutive cardiac cycles. The global LV systolic function was expressed as fractional shortening (FS%).

### 4.3. Morphometric Analysis to Determine the Area at Risk

The area at risk (AAR) was determined as previously described [27]. Briefly, three days after surgery, the LAD was re-tied with the suture left in its original position and 1 mL 1% Evan’s blue (Sigma Aldrich, Saint Louis, MO, USA) was injected in the inferior cava vein to identify the myocardial AAR as unstained. Next, the heart, arrested in diastole, was excised and cut in transversal and parallel slices about 2 mm thick. LV area and the AAR, (expressed as percentage of the LV) from each slice were measured using Image J software (open source image processing program).

### 4.4. Serum and Tissue Thyroid Hormone Levels

Two mL of blood were collected from the femoral vein both 3–5 days before (basal) and 3 days after LAD occlusion (terminal endpoint) in anesthetized animals. Serum free T3 (FT3) and free T4 (FT4) were assayed as previously described [27].

### 4.5. Mitochondria Isolation

Mitochondria were purified from LV fresh tissue according to the manufacturer’s protocol (MITO-ISO1; Sigma Aldrich, Saint Louis, MO, USA) and as previously described [26]. Briefly, cardiac tissue was homogenized in buffer containing 10 mM 4-(2-Hydroxyethyl)piperazine-1-ethanesulfonic acid, *N*-(2-Hydroxyethyl)piperazine-*N*′-(2-ethanesulfonic acid) (HEPES, Sigma Aldrich), 200 mM mannitol (Sigma Aldrich), 70 mM sucrose and 1mM Ethylene glycol-bis(2-aminoethylether)-*N,N,N′,N′*-tetraacetic acid(EGTA) (PH 7.5, Sigma Aldrich) and centrifuged at 2000× *g* at 4 °C for 5 min. The supernatant was collected and centrifuged at 11,000× *g* at 4 °C for 20 min. The pellet was suspended in storage buffer at pH 7.5 containing 10 mM HEPES, 250 mM sucrose (Sigma Aldrich), 1 mM ATP (Sigma Aldrich), 0.08 mM ADP (Sigma Aldrich), 5 mM sodium succinate (Sigma Aldrich), 2 mM K_2_HPO_4_ (Sigma Aldrich) and 1 mM DTT (Sigma Aldrich) and stored at −80 °C until use. An aliquot of the suspended pellet was assayed for protein content with the Pierce bicinchoninic protein assay kit (Sigma Aldrich).

### 4.6. Mitochondrial Enzyme Activity Assays

Mitochondrial function was expressed as the ratio between the activity of the cytochrome c oxidase-1 (CcOX-1) and that of citrate synthase (CS) to normalize for mitochondrial mass. Enzyme activity was assessed in 1mL cuvette by using spectrophotometric assay kit according to the manufacturer’s protocols (CYTOC-OX1 and CS0720, Sigma Aldrich) and as previously described [27]. All assays were performed in triplicate.

### 4.7. Measurements of ATP Production in Isolated Mitochondria

ATP synthesis rates were measured in mitochondrial fractions with the ATP Determination Kit A22066 (Thermo Scientific, Waltham, MA, USA) as previously described [27]. The assays were performed in triplicate in 96 well plate in a volume of 150 μL containing10 μg mitochondria protein, 0.25 M sucrose, 50 mM HEPES, 2 mM MgCl_2_, 1 mM EGTA, 10 mM KH_2_PO_4_, 1 mM pyruvate, 1 mM malate. 1 mM ATP-free ADP and a solution of 0.5 mM luciferin and 0.25 μg/mL luciferase were added with the injectors integrated in the plate reader (Infinite M200 PRO, TECAN, Männedorf, Switzerland). The slope of luminescence increase was determined in the first 48 s after injection of luciferase reagent and ADP, and it was converted to ATP concentration using a standard curve according to the manufacturer’s instruction.

### 4.8. Proteomics Sample Pre-Processing

For proteomic analyses the crude mitochondrial fraction was further purified on Percoll gradient as previously described with some modification [70]. Briefly, mitochondria were resuspended in 1 mL of 15% Percoll and layered on preformed gradient consisting of 22% Percoll (3 mL) layered over 50% Percoll (1 mL). Following centrifugation of the gradient at 90,000× *g* for 40 min, the mitochondrial fraction that accumulated at the lower interface (between the 50% and 22% Percoll layers) was collected and diluted with PBS (1:8). After 2 washing step in PBS, the pellet was lysed in TRIS HCl 5 mM pH = 8.1, acetonitrile 10% (Romil, Cambridge, UK) and protease inhibitor and sonicated in ice. Protein concentration was determined by bicinchoninic acid assay (Pierce, Thermo Scientific, Waltham, MA, USA). About 20 µg of protein were diluted in Ammonium Bicarbonate 25 mM, reduced with dithiothreitol DTT 5 mM at 80 °C for 30 min and alkylated using iodoacetamide 10 mM at 37 °C for 20 min. Digestion was performed incubating overnight with 1:100 trypsin (Roche, Basel, Switzerland):substrate at 37 °C. Peptides solution was then loaded on a C18 cartridge in order to eliminate debris and filtered with 0.22 μm filter. Peptide mix was diluted to 100 µL by 2% ACN/0.1% FA.

### 4.9. nanoLC-MS/MS SWATH-Based Analysis

Chromatographic separation of peptides was performed using a nano-HPLC system (Eksigent, ABSciex, Washington, DC, USA). The loading pump pre-concentrated the sample in a pre-column cartridge (PepMap-100 C18 5 µm 100 A, 0.1 × 20 mm, Thermo Scientific, Waltham, MA, USA) and then separated in a C18 PepMap-100 column (3 µm, 75 µm × 250 mm, Thermo Scientific) at a flow rate of 300 nL·min^−1^. Runs were performed with eluent A (Ultrapure water, 0.1% Formic acid) under 60 min linear gradient from 5% to 40% of eluent B (Acetonitrile, 0.1% Formic acid) followed by 10 min of a purge step and 20 min re-equilibration step. Peptides eluted from chromatography were directly processed using 5600 TripleTOF™ mass spectrometer (ABSciex) equipped with a DuoSpray™ ion source (ABSciex). Data were acquired using the new Sequential Window Acquisition of all THeoretical Mass Spectra (SWATH™) method for shotgun data independent MRM quantification. For library, MS/MS data were processed with ProteinPilot™ Software (ABSciex). The false discovery rate (FDR) analysis was done using the integrated tools in ProteinPilot software (ABSciex, Washington, DC, USA) and a confidence level of 95% was set.

The label free statistical comparative analysis was performed using PeakView4.5 Software (ABSciex) with MS/MS(ALL) with SWATH™ Acquisition MicroApp 2.0 and MarkerViewTM (ABSciex). Retention time alignment was obtained using selected peptides from top score protein. Processing settings were: 7 peptides per protein, 7 transitions per peptide, 92% peptide confidence (according to Paragon algorithm result) and 5% FDR; XIC (Extracted-Ion Chromatogram) options: extraction window 10 min, width 50 ppm and 0.1 Da. Normalization of the sample content was done using a global normalization of profiles (based on total protein content). Principal Component Analysis (PCA) was performed in order to evidence groupings among the data set. SWATH strategy generate time-resolved fragment ion spectra sufficiently specific to confidently identify query peptides which are quantified with a consistency and accuracy comparable with that of selected reaction monitoring, the gold standard proteomic quantification method [71]. This means that differential expression analysis of SWATH data allows the profiling of disease-related proteomes with a high degree of reproducibility and confidence providing self-validated data [72].

### 4.10. Statistical Analysis

Results are given as mean + SEM unless otherwise stated. Since the post IR circulating free T3 (FT3) levels have been used to allocate rats to different groups, we verified the distribution of this parameter for normality (Kolmogorov-Smirnov) before inferential statistic analysis. Differences among the three groups of rats were analyzed by a one-way ANOVA followed by a Bonferroni test once normality had been proven (Kolmogorov-Smirnov test). Differences were considered statistically significant at a value of *p* < 0.05. For proteomic analyses, a non-parametric test (Kruskall-Wallis) was run considering all the three groups. Thereafter, a Mann-Whitney *U*-test (adjusting the α-level by Bonferroni inequality) was used to check differences between groups two by two (differences were considered statistically significant at a value of *p* < 0.017). The significant proteins resulting from the Mann-Whitney *U*-test comparison between IR-NT3 and IR-LT3 (*n* = 82) were used as input dataset for IPA and networks were created for the most significant linked diseases and functions. Additionally, proteins involved in TCA cycle pathway were shown (Figure 2B).

## Supplementary Materials

Supplementary materials can be found at http://www.mdpi.com/1422-0067/16/11/25973/s1.

## Abbreviations

Acaa2 = 3-ketoacyl-CoA thiolase.
Acadl = Long-chain specific acyl-CoA dehydrogenase.
Acadm = Acetyl-Coenzyme A dehydrogenase, medium chain.
Acads = Acetyl-Coenzyme A dehydrogenase, short chain.
Acat1 = Acetyl-CoA acetyltransferase.
Aco2 = Aconitate hydratase.
Acot2 = Acyl-CoA thioesterase 2.
Acsf2 = Acyl-CoA synthetase family member 2.
Aldh2 = Aldehyde dehydrogenase.
Aldh6a1 = Aldehyde dehydrogenase family 6, isoform CRA.
Ckmt2 = Creatine kinase mitochondrial.
Cpt1b = Carnitine *O*-palmitoyltransferase 1.
Cryab = α-crystallin B chain.
Cs = Citrate synthase.
Decr1 = 2,4-dienoyl CoA reductase 1.
Dld = Dihydrolipoyl dehydrogenase.
Dlst = Dihydrolipoamide *S*-succinyltransferase.
Ech1 = δ(3,5)-δ(2,4)-dienoyl-CoA isomerase.
Echs1 = Enoyl-CoA hydratase.
Eci1 = Dodecenoyl-Coenzyme A δ isomerase (3,2 trans-enoyl-Coenzyme A isomerase).
Ecl1 = Extender of the chronological lifespan protein 1.
Ecl2 = Extender of the chronological lifespan protein 2.
Fabp3 = Fatty acid-binding protein.
Fh = Fumarate hydratase 1.
Got2 = Aspartate aminotransferase.
Hadh = Hydroxyacyl-coenzyme A dehydrogenase.
Hibadh = 3-hydroxyisobutyrate dehydrogenase.
Hsc70 = Heat shock cognate 71 kDa.
Hspb6 = Heat shock protein α-crystallin-related-B6.
Hsp90ab1 = Heat shock protein HSP 90-α class B, member 1.
Hspb1 = Heat shock 27 kDa protein 1.
Hspd1 = Heat shock protein 60 kDa.
Hspe1 = Heat shock 10kDa protein 1.
Idh2 = Isocitrate dehydrogenase (NADP).
Ivd = Isovaleryl-CoA dehydrogenase.
Ldhb = l-lactate dehydrogenase B chain.
LOC683884 = Protein Acot13.
Lrpprc = Leucine-rich PPR.
Ndufab1 = NADH dehydrogenase (ubiquinone) 1, α/β subcomplex, 1.
Macrod1 = *O*-acetyl-ADP-ribose deacetylase MACROD1.
Mdh2 = Malate dehydrogenase.
MOR4B8 = Pyruvate kinase.
Ogdh = 2-oxoglutarate dehydrogenase.
Oxct1 = Succinyl-CoA:3-ketoacid coenzyme A transferase 1.
Pdha1/1 = Protein Pdha1/1.
Pdk1 = Pyruvate dehydrogenase kinase 1.
Pdk2 = Pyruvate dehydrogenase kinase 1.
Pepb1 = Phosphatidylethanolamine-binding protein 1.
Pgk1 = Phosphoglycerate kinase 1.
Prdx2 = Peroxiredoxin-2.
Prdx5 = Peroxiredoxin-5.
Pygb = Glycogen phosphorylase.
Rps3 = 40 S ribosomal protein S3.
Sdha = Succinate dehydrogenase (ubiquinone) flavoprotein subunit.
Sdhb = Succinate dehydrogenase (ubiquinone) iron-sulfur subunit.
Sdhc = Succinate dehydrogenase complex, subunit C, integral membrane protein.
Slc25a3 = Phosphate carrier protein.
Sod1 = Superoxide dismutase [Cu-Zn].
Sod2 = Superoxide dismutase [Cu-Zn].
Sucla2 = succinyl-CoA synthetase.
Suclg1 = Succinyl-CoA synthetase (ADP/GDP-forming) subunit α.
Tgm2 = transglutaminase 2.
Vdac1 = Voltage-dependent anion-selective channel protein 1.
Vdac2 = Voltage-dependent anion-selective channel protein 2.
