# Supplementary material for: Low T3 State Is Correlated with Cardiac Mitochondrial Impairments after Ischemia Reperfusion Injury: Evidence from a Proteomic Approach
Source: Int J Mol Sci. 2015 Nov 6;16(11):26687–705. doi: 10.3390/ijms161125973 (PMC4661832; doi:10.3390/ijms161125973)
Supplement: Supplementary file 1 [file ijms-16-25973-s001.pdf]

International Journal of
Molecular Sciences

MDPI

Supplementary Information

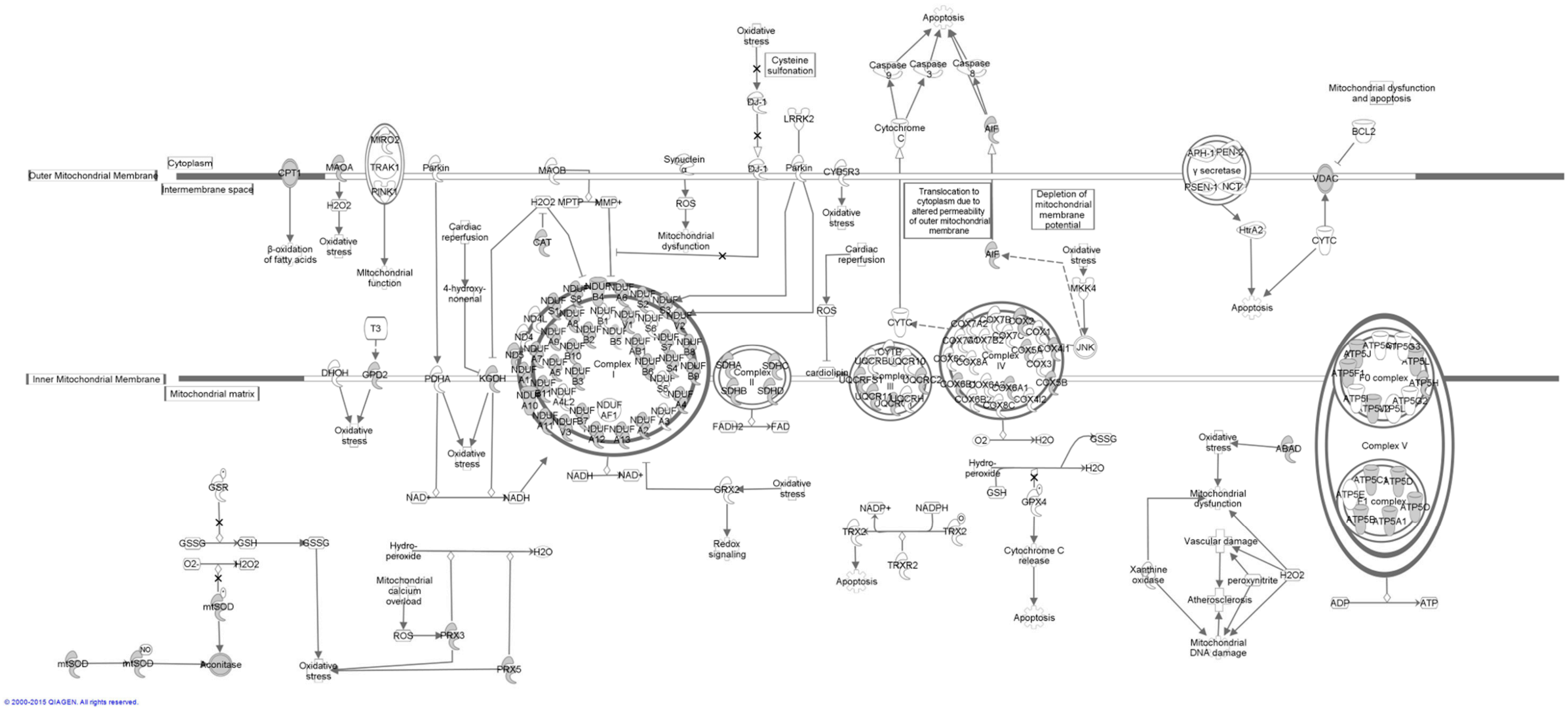

Figure S1. IPA representation of mitochondrial pathways. The complete list of identified proteins was submitted to IPA. Almost all the mitochondrial functional proteins were mapped (shown in grey).

Table S1. Differentially expressed proteins involved in cell death and mitochondrial quality control in response to stress.

Accession Number	Gene Name	Protein Name	Function, Process	IR-LT3S (FC)	IR-NT3 (FC)
Mitochondria-mediated cell death:					
G3V741	*Slc25a3*	Phosphate carrier protein	Mitochondria permeability transitinon (cell death)	NS	−2.2 *
Q9WVJ6	*Tgm2*	Protein Tgm2	Clearance of damaged mitochondria	7.0	39 *
Q9Z2L0	*Vdac1*	Voltage-dependent anion-selective channel protein 1	Apoptosis, necrosis	3.4	NS *
P81155	*Vdac2*	Voltage-dependent anion-selective channel protein 2	Apoptosis, necrosis	3.4	1.80
Q9R1Z0	*Vdac3*	Voltage-dependent anion-selective channel protein 3	Apoptosis, necrosis	3.5	2.05
Mitochondria quality control:					
G3V913	*Hspb1*	Heat shock 27 kDa protein 1	Chaperone, negative regulation of oxidative stress	42	67 *
P63018	*Hspa8*	Heat shock cognate 71 kDa	Chaperone-mediated protein folding	NS	20 *
P97541	*Hspb6*	Heat shock protein β-6	Chaperone, protein homodimerization	NS	3.1 *
P34058	*Hsp90ab1*	Heat shock protein HSP 90-β	Chaperone, maturation, structural maintenance and proper regulation of target proteins	7.5	24 *
P63039	*Hspd1*	Heat shock protein 60 kDa	Mitochondrial protein import and macromolecular assembly	4.4	4.1
P97576	*Grpel1*	GrpE protein homolog 1	Proteins translocation from the inner membrane into the mitochondrial matrix	4.9	7.3
P23928	*Cryab*	α-crystallin B chain	Chaperone-like activity, preventing aggregation of various proteins under stress conditions	14	27 *
Q5XIM0	*Bcs1l*	BCS1-like (Yeast)	Chaperone-like protein essential for mitochondrial complex assemby	4.2	5.3
Q5BJQ0	*Adck3*	Chaperone activity of bc1 complex-like	Chaperone-like. Proper conformation and functioning of protein complexes in the respiratory chain	5.6	6.2
D4AC73	*Chchd7*	Protein Chchd7	Assembly of mitochondrial respiratory complex COX23 Cytochrome C Oxidase Assembly Homolog	7.3	1.4 *
P85834	*Tufm*	Elongation factor Tu	Mitochondrial protein biosynthesis	5.1	3.7 *
Q68FT1	*Coq9*	Ubiquinone biosynthesis protein	Cofactor biosynthesis	2.4	8.0 *
P62076	*Timm13*	Mitochondrial import inner membrane translocase subunit Tim13	Chaperone-like, protein folding and guiding through the mitochondrial intermembrane space	NS	1.3 *
P62909	*Rps3*	40S ribosomal protein S3	Repair of damaged DNA	8.7	28 *
G3V8V6	*Macrod1*	*O*-acetyl-ADP-ribose deacetylase MACROD1	Cellular response to DNA damage stimulus	7.3	15 *
P31044	*Pebp1*	Phosphatidylethanolamine-binding protein 1	Serine protease inhibitor, responce to oxidative stress and wounding	3.7	14 *
F1LM33	*Lrpprc*	Leucine-rich PPR	Translation or stability of mitochondrially encoded cytochrome c oxidase subunits	2.9	4.4
P13437	*Acaa2*	3-ketoacyl-CoA thiolase	Abolishes mitochondrial damage and BNIP3-mediated apoptosis	13	15

Table S1. *Cont.*

Accession Number	Gene Name	Protein Name	Function, Process	IR-LT3S (FC)	IR-NT3 (FC)
Antioxidant defence:					
G3V7J0	*Aldh6a1*	Aldehyde dehydrogenase family 6, isoform CRA	Aldehyde clearance, prevents aldehyde mediated oxidative stress	4.2	10 *
F1LN88	*Aldh2*	Aldehyde dehydrogenase	Aldehyde clearance, prevents aldehyde mediated oxidative stress	NS	9.6 *
P35704	*Prdx2*	Peroxiredoxin-2	Antioxidant	NS	19 *
Q9R063	*Prdx5*	Peroxiredoxin-5	Antioxidant	NS	39 *
P07632	*Sod1*	Superoxide dismutase [Cu-Zn]	Antioxidant	NS	8.8 *
P07895	*Sod2*	Superoxide dismutase [Mn]	Antioxidant	NS	12 *

Comparison among groups was performed as described in statistical analysis. FC = fold change with respect to sham group; NS = not significantly different from sham group; * p *vs.* IR-LT3S < 0.017.

Table S2. Differentially expressed proteins involved in electron transport and oxidative phosphorilation.

Accession Number	Gene Name	Protein Name	Function, Process	IR-LT3S (FC)	IR-NT3 (FC)
P11608	*Mt-atp8*	ATP synthase protein 8	ATP synthesis complex V	−55	−40
P15999	*Atp5a1*	ATP synthase subunit α	ATP synthesis complex V	−4.5	−5.2
G3V6D3	*Atp5b*	ATP synthase subunit β	ATP synthesis complex V	−3.0	−2.7
P31399	*Atp5h*	ATP synthase subunit d	ATP synthesis complex V	−10	−15
G3V7Y3	*Atp5d*	ATP synthase subunit δ	ATP synthesis complex V	−6.4	−7.7
P29419	*Atp5i*	ATP synthase subunit e	ATP synthesis complex V	−9.2	−13
F7FFJ9	*Atp5c1*	ATP synthase subunit γ	ATP synthesis complex V	−1.92	−2.5
Q06647	*Atp5o*	ATP synthase subunit O	ATP synthesis complex V	−3.6	−3.3
P21571	*Atp5j*	ATP synthase-coupling factor 6	ATP synthesis complex V	−4.4	−16
Q68FY0	*Uqcrc1*	Cytochrome b-c1 complex subunit 1	Electron transport complex III	−3.1	−4.7 *
P32551	*Uqcrc2*	Cytochrome b-c1 complex subunit 2	Electron transport complex III	−2.0	−2.4
Q5M9I5	*Uqcrh*	Cytochrome b-c1 complex subunit 6	Electron transport complex III	1.30	−15 *
Q7TQ16	*Uqcrq*	Cytochrome b-c1 complex subunit 8	Electron transport complex III	−4.3	−3.9
P20788	*Uqcrfs1*	Cytochrome b-c1 complex subunit Rieske	Electron transport complex III	−5.6	−6.0
Q8SEZ5	*Mt-co2*	Cytochrome c oxidase subunit 2	Electron transport complex IV	NS	−3.6 *
P10888	*Cox4i1*	Cytochrome c oxidase subunit 4 isoform 1	Electron transport complex IV	−1.7	−6.2 *
P11240	*Cox5a*	Cytochrome c oxidase subunit 5A	Electron transport complex IV	NS	−3.4 *

Table S2. *Cont.*

Accession Number	Gene Name	Protein Name	Function, Process	IR-LT3S (FC)	IR-NT3 (FC)
D3ZD09	*Cox6b1*	Cytochrome c oxidase subunit 6B1	Electron transport complex IV	−3.6	−5.6
P11951	*Cox6c2*	Cytochrome c oxidase subunit 6C-2	Electron transport complex IV	−3.3	−8.8 *
P35171	*Cox7a2*	Cytochrome c oxidase subunit 7A2	Electron transport complex IV	−4.0	−4.4
D3ZYX8	*Cox7a2l*	Cytochrome c oxidase subunit VIIa polypeptide 2 like	Electron transport complex IV	−27	−12.5
D3ZFQ8	*Cyc1*	Cytochrome c-1 (Predicted), isoform CRA_b	Electron carrier protein	NS	−3.8 *
Q66HF3	*Etfdh*	Electron transfer flavoprotein-ubiquinone oxidoreductase	Electron carrier protein	NS	−2.3 *
Q5RK08	*Gbas*	Glioblastoma amplified sequence	Oxidative phosphorylation	9.00	6.5 *
F1LXA0	*Ndufa12*	NADH dehydrogenase (Ubiquinone) 1 α subcomplex, 12	Electron transport complex I	−2.4	−2.7
B2RYS8	*Ndufb8*	NADH dehydrogenase (Ubiquinone) 1 β subcomplex 8	Electron transport complex I	1.24	−5.4 *
D4A565	*Ndufb5*	NADH dehydrogenase (Ubiquinone) 1 β subcomplex, 5	Electron transport complex I	NS	−2.1 *
D3ZZ21	*Ndufb6*	NADH dehydrogenase (Ubiquinone) 1 β subcomplex, 6	Electron transport complex I	NS	−4.7 *
D3ZLT1	*Ndufb7*	NADH dehydrogenase (Ubiquinone) 1 β subcomplex, 7	Electron transport complex I	−1.71	−1.29
B2RYW3	*Ndufb9*	NADH dehydrogenase (Ubiquinone) 1 β subcomplex, 9	Electron transport complex I	NS	−6.2 *
D3ZG43	*Ndufs3*	NADH dehydrogenase (Ubiquinone) Fe-S protein 3	Electron transport complex I	1.73	−2.2
Q5RJN0	*Ndufs7*	NADH dehydrogenase (Ubiquinone) Fe-S protein 7	Electron transport complex I	2.07	NS *
B0BNE6	*Ndufs8*	NADH dehydrogenase (Ubiquinone) Fe-S protein 8	Electron transport complex I	4.0	NS *
Q5XIH3	*Ndufv1*	NADH dehydrogenase (Ubiquinone) flavoprotein 1	Electron transport complex I	1.43	−2.2 *
Q561S0	*Ndufa10*	NADH dehydrogenase (Ubiquinone) 1 α subcomplex subunit 10	Electron transport complex I	−3.1	−1.8 *
D3ZS58	*Ndufa2*	NADH dehydrogenase (Ubiquinone) 1 α subcomplex subunit 2	Electron transport complex I	1.37	−4.2 *
Q63362	*Ndufa5*	NADH dehydrogenase (Ubiquinone) 1 α subcomplex subunit 5	Electron transport complex I	2.7	−2.7 *
Q5BK63	*Ndufa9*	NADH dehydrogenase (Ubiquinone) 1 α subcomplex subunit 9	Electron transport complex I	2.8	NS *
Q5PQZ9	*Ndufc2*	NADH dehydrogenase (Ubiquinone) 1 subunit C2	Electron transport complex I	−25	−29
P19234	*Ndufv2*	NADH dehydrogenase (Ubiquinone) flavoprotein 2	Electron transport complex I	NS	−3.1 *
Q641Y2	*Ndufs2*	NADH dehydrogenase (Ubiquinone) iron-sulfur protein 2	Electron transport complex I	1.97	−1.4 *
Q5XIF3	*Ndufs4*	NADH dehydrogenase (Ubiquinone) iron-sulfur protein 4	Electron transport complex I	3.30	NS *
Q66HF1	*Ndufs1*	NADH-ubiquinone oxidoreductase 75 kDa subunit	Electron transport complex I	1.44	−3.1
Q8SEZ0	*Mt-nd5*	NADH-ubiquinone oxidoreductase chain 5	Electron transport complex I	−3.0	−5.4
A9UMV9	*Ndufa7*	Ndufa7 protein	Electron transport complex I	−7.9	−5.2
D4A5L9	*LOC679794*	Protein LOC679794	Electron carrier protein	−11	NS *
D4A4P3	*Ndufa3*	Protein LOC100361144	Electron carrier protein	2.14	−6.2 *
F1LPG5	*Ndufa4*	Protein LOC688963	Electron carrier protein	−2.4	−2.4

Comparison among groups was performed as described in statistical analysis. FC = fold change with respect to sham group; NS = not significantly different from sham group; * p *vs.* IR-LT3S < 0.017.

Table S3. Differentially expressed proteins involved in pyruvate metabolism and tricarboxylic acid cycle.

Accession Number	Gene Name	Protein Name	Function, Process	IR-LT3S (FC)	IR-NT3 (FC)
P00507	*Got2*	Aspartate aminotransferase	Pre TCA, anaplerotic reaction	NS	10 *
P38718	*Mpc2*	Mitochondrial pyruvate carrier 2	Mitochondrial pyruvate transport (pre TCA)	16	25
D4A5G8	*Pdha1l1*	Protein Pdha1/1	Conversion of pyruvate to acetyl-CoA (pre TCA)	2.8	5.0 *
P49432	*Pdhb*	Pyruvate dehydrogenase E1 component subunit β	Conversion of pyruvate to acetyl-CoA (pre TCA)	1.7	3.6
F1MA54	*Pdk1*	Pyruvate dehydrogenase (acetyl-transferring) kinase isozyme 1	Conversion of pyruvate to acetyl-CoA (pre TCA)	11	15 *
Q64536	*Pdk2*	Pyruvate dehydrogenase (acetyl-transferring) kinase isozyme 2	Conversion of pyruvate to acetyl-CoA (pre TCA)	6.7	13 *
Q8VHF5	*Cs*	Citrate synthase	TCA	NS	7.2 *
Q9ER34	*Aco2*	Aconitate hydratase	TCA	2.0	4.9 *
F1LNF7	*Idh3a*	Isocitrate dehydrogenase (NAD) subunit α	TCA	4.2	4.5
Q68FX0	*Idh3B*	Isocitrate dehydrogenase (NAD) subunit β	TCA	5.1	7.0
P56574	*Idh2*	Isocitrate dehydrogenase (NADP)	TCA	5.8	10 *
Q5XIJ3	*Idh3g*	Isocitrate dehydrogenase 3 (NAD), γ	TCA	10	15
Q5XI78	*Ogdh*	2-oxoglutarate dehydrogenase	TCA	N.S	4.0 *
G3V6P2	*Dlst*	Dihydrolipoamide S-succinyltransferase	TCA, (E2 component of 2-oxo-glutarate complex)	NS	2.3 *
Q6P6R2	*Dld*	Dihydrolipoyl dehydrogenase	TCA, (E3 component of 2-oxo-glutarate complex)	−1.8	4.7 *
F1LM47	*Sucla2*	Protein Sucla2	TCA (succinyl-CoA synthetase)	12	18 *
P13086	*Suclg1*	Succinyl-CoA synthetase (ADP/GDP-forming) subunit α	TCA	3.1	5.3 *
Q641Z9	*Sdhc*	Succinate dehydrogenase complex, subunit c, integral membrane	TCA (succinate dehydrogenase activity)	1.6	1.7
Q6PCT8	*Sdhd*	Succinate dehydrogenase (ubiquinone) cytochrome b small subunit	TCA	3.0	3.3
Q920L2	*Sdha*	Succinate dehydrogenase (ubiquinone) flavoprotein subunit	TCA	1.4	−1.4 *
P21913	*Sdhb*	Succinate dehydrogenase (ubiquinone) iron-sulfur subunit	TCA	1.4	NS *
Q5M964	*Fh*	Fumarate hydratase 1	TCA	NS	3.4 *
P04636	*Mdh2*	Malate dehydrogenase	TCA	−7.7	NS *

TCA = tricarboxylic acid cycle. Comparison among groups was performed as described in statistical analysis. FC = fold change with respect to sham group; NS = not significantly different from sham group; * p *vs.* IR-LT3S < 0.017.

Table S4. Differentially expressed proteins involved in fatty acid transport, biosynthesis and oxidation.

Accession Number	Gene Name	Protein Name	Function, Process	IR-LT3S (FC)	IR-NT3 (FC)
G3V734	*Decr1*	2,4-dienoyl CoA reductase 1	Auxiliary enzyme of β oxidation	2.6	13 *
D3ZA93	*Acot13*	Protein Acot13	Regulate intracellular levels of acyl-CoAs, free fatty acids and CoASH	6.1	10 *
G3V796	*Acadm*	Acetyl-Coenzyme A dehydrogenase, medium chain	Fatty acid β oxidation	3.5	5.9 *
Q6IMX3	*Acads*	Acetyl-Coenzyme A dehydrogenase, short chain	Fatty acid β oxidation	4.8	6.1 *
D3ZF13	*Ndufab1*	Acyl carrier protein	Fatty acid biosynthesis	1.4	−3.4 *
Q499N5	*Acsf2*	Acyl-CoA synthetase family member 2	Fatty acid metabolism	7.3	15 *
Q6IMX8	*Acot2*	Acyl-CoA thioesterase 2	Increases the utilization of fatty acid substrate	NS	7.1 *
Q5M9H2	*Acadvl*	Acyl-Coenzyme A dehydrogenase, very long chain	Fatty acid β oxidation	4.0	2.4
Q63704	*Cpt1b*	Carnitine *O*-palmitoyltransferase 1	Fatty acid β oxidation	2.9	1.6 *
P18886	*Cpt2*	Carnitine *O*-palmitoyltransferase 2	Fatty acid β oxidation	3.0	3.0
Q62651	*Ech1*	Δ(3,5)-Δ(2,4)-dienoyl-CoA isomerase	Lipid metabolism, fatty acid β oxidation	NS	6.2 *
Q68G41	*Eci1*	Dodecenoyl-Coenzyme A δ isomerase (3,2 *trans*-enoyl-Coenzyme A isomerase)	Fatty acid β oxidation	NS	2.1 *
P13803	*Etfa*	Electron transfer flavoprotein subunit α	Lipid metabolism	4.04	3.76
Q68FU3	*Etfb*	Electron transfer flavoprotein subunit β	Lipid metabolism	2.30	2.72
Q5XIC0	*Eci2*	Enoyl-CoA δ isomerase 2	Fatty acid β oxidation	3.7	6.0
P14604	*Echs1*	Enoyl-CoA hydratase	Fatty acid β oxidation	NS	6.4 *
P07483	*Fabp3*	Fatty acid-binding protein	Long chain fatty acid transport, fatty acid β oxidation	NS	49 *
Q9WVK7	*Hadh*	Hydroxyacyl-coenzyme A dehydrogenase	Lipid metabolism; fatty acid β-oxidation	NS	22 *
P15650	*Acadl*	Long-chain specific acyl-CoA dehydrogenase	Fatty acid β oxidation	2.2	5.0 *
P14882	*Pcca*	Propionyl-CoA carboxylase α chain	Lipid metabolism	5.2	5.2
Q68FZ8	*Pccb*	Propionyl coenzyme A carboxylase, β polypeptide	Lipid metabolism	2.8	4.5
M0R7V3	*Apoo*	Protein Apoo	Lipid transport	−1.4	−3.1

Comparison among groups was performed as described in statistical analysis. FC = fold change with respect to sham group; NS = not significantly different from sham group; * p *vs.* IR-LT3S < 0.017.

Table S5. Differentially expressed proteins involved in various metabolic process and energy homeostasis.

Accession Number	Gene Name	Protein Name	Function, Pathway	IR-LT3S (FC)	IR-NT3 (FC)
Carbohydrate, amino acid and keton body metabolism:					
P42123	*Ldhb*	L-lactate dehydrogenase B chain	Glycolysis, lactate clearance	NS	35 *
M0R4B8	*N/A*	Pyruvate kinase	Glycolysis	2.4	30 *
P16617	*Pgk1*	Phosphoglycerate kinase 1	Glycolysis	3.8	22 *
G3V6Y6	*Pygb*	Glycogen phosphorylase	Carbohydrate metabolism	12	40 *
B2GV15	*Dbt*	Dihydrolipoamide branched chain transacylase E2	Amino acid catabolic process	6.7	5.5
B1WBN3	*Bckdha*	2-oxoisovalerate dehydrogenase subunit α	Amino acid catabolic process	5.8	5.6
P29266	*Hibadh*	3-hydroxyisobutyrate dehydrogenase	Amino acid degradation	3.2	6.3 *
P12007	*Ivd*	Isovaleryl-CoA dehydrogenase	Amino acid degradation	3.0	7.3 *
Q5XIT9	*Mccc2*	Methylcrotonoyl-CoA carboxylase β chain	Amino acid degradation	3.4	4.5
F1LP30	*Mccc1*	Methylcrotonoyl-CoA carboxylase subunit α	Amino acid degradation	9.7	7.9
F1LQZ0	*Tmem65*	Protein Tmem65	Amino acid catabolic process	3.2	2.1
B2GV06	*Oxct1*	Succinyl-CoA:3-ketoacid coenzyme A transferase 1	Keton body catabolism	NS	4.9 *
P17764	*Acat1*	Acetyl-CoA acetyltransferase	Ketone body metabolism, lipid metabolism	−1.9	1.6 *
Energy homeostasis:					
Q9WUS0	*Ak4*	Adenylate kinase 4	Homeostasis of cellular nucleotides, cellular energy homeostasis	1.9	2.3
M0RC66	*Ak1*	Adenylate kinase isoenzyme 1 (Fragment)	Homeostasis of cellular nucleotides, cellular energy homeostasis	27	50
P09605	*Ckmt2*	Creatine kinase S-type	Energy transduction in tissues with large, fluctuating energy	−6.1	−2.7 *

Comparison among groups was performed as described in statistical analysis. FC = fold change with respect to sham group; NS = not significantly different from sham group; * p *vs.* IR-LT3S < 0.017.
